# *Tubulator*: an automated approach to analysis of t-tubule and dyadic organization in cardiomyocytes

**DOI:** 10.1098/rstb.2021.0468

**Published:** 2022-11-21

**Authors:** Michael Frisk, Per Andreas Norseng, Emil Knut Stenersen Espe, William E. Louch

**Affiliations:** ^1^ Institute for Experimental Medical Research, Oslo University Hospital and University of Oslo, 0424 Oslo, Norway; ^2^ K.G. Jebsen Cardiac Research Center and Center for Heart Failure Research, University of Oslo, 0316 Oslo, Norway

**Keywords:** transverse tubules, dyadic structure, EC-coupling, cardiac disease, analysis software

## Abstract

During cardiac disease, t-tubules and dyads are remodelled and disrupted within cardiomyocytes, thereby reducing cardiac performance. Given the pathological implications of such dyadic remodelling, robust and versatile tools for characterizing these sub-cellular structures are needed. While analysis programs for continuous and regular structures such as rodent ventricular t-tubules are available, at least in two dimensions, these approaches are less appropriate for assessment of more irregular structures, such as dyadic proteins and non-rodent t-tubules. Here, we demonstrate versatile, easy-to-use software that performs such analyses. This software, called *Tubulator*, enables automated analysis of t-tubules and dyadic proteins alike, in both tissue sections and isolated myocytes. The program measures densities of subcellular structures and proteins in individual cells, quantifies their distribution into transversely and longitudinally oriented elements, and supports detailed co-localization analyses. Importantly, *Tubulator* provides tools for three-dimensional assessment and rendering of image stacks, extending examinations from the single plane to the whole-myocyte level. To provide insight into the consequences of dyadic organization for synchrony of Ca^2+^ handling, *Tubulator* also creates ‘distance maps', by calculating the distance from all cytosolic positions to the nearest t-tubule and/or dyad. In conclusion, this freely accessible program provides detailed automated analysis of the three-dimensional nature of dyadic and t-tubular structures.

This article is part of the theme issue ‘The cardiomyocyte: new revelations on the interplay between architecture and function in growth, health, and disease’.

## 1. Introduction

In cardiomyocytes, invaginations of the sarcolemmal membrane form a complex system, known as the t-tubular network. Although t-tubules are primarily oriented transversely across the cardiomyocyte, a substantial fraction of ‘axial' or ‘longitudinal' tubules may be oriented along the long axis of the cell. Both transverse and longitudinal elements form dyadic junctions with the sarcoplasmic reticulum (SR). This close localization of the dyadic membranes enables the opening of L-type Ca^2+^ channels in t-tubules to trigger Ca^2+^ release from opposing ryanodine receptors in the SR, eliciting contraction. A high density of well-organized t-tubules and dyads facilitate synchronized Ca^2+^ release across the entire myocyte and efficient triggering of contraction [1–5]. T-tubule and dyadic organization vary between species and even within chambers of the individual heart. Additionally, several studies have shown that these structures are subject to considerable plasticity [2,5]. For instance, dyads are disrupted and degraded during cardiac diseases such as heart failure, leading to impaired cardiomyocyte Ca^2+^ handling, and hence reduced whole-heart contractility [2]. Given the importance of proper dyadic function, and the implications of remodelling during disease, t-tubules and dyads have been subject to increasing research interest during recent years. Therefore, robust and versatile analysis tools are needed.

First and foremost, assessing dyadic remodelling requires quality imaging of cardiomyocyte structures, acquired with optimized labelling specificity and intensity. Once recorded, dyadic structure has traditionally been quantified in two ways; firstly, by density analyses (e.g. fraction of cell covered by structure of interest) in binarized images, and secondly, by regularity analysis using fast Fourier transformation (FFT). While these approaches are practical and easily implemented, both methods are sensitive to variations in image quality and pixel intensity. A common recurring problem when analysing density is that automated thresholding algorithms are sensitive to the overall t-tubule signal, thus biasing threshold settings towards lower values in cells with fewer t-tubules. By comparison, FFT analyses are, in addition to being susceptible to intensity-driven bias, also sensitive to cell size and require regular structures to yield readily interpretable results. An automated ImageJ plugin using these methods was previously published [6], but it was not until the Song laboratory created a plugin called *AutoTT* that some of these shortcomings were circumvented [7]. Their plugin automatically normalizes fluorescent intensity to allow for better comparison between images with variable clarity and brightness of t-tubule staining. It also calculates parameters describing t-tubule structural features and density with better accuracy than previous attempts. Although this program functions well on isolated cardiomyocytes, it does not allow analysis of multi-cell preparations such as whole-heart *in situ* preparations and cryosections. In addition, the plugin relies on continuous structures, thereby limiting its use on tissue where t-tubules or dyadic proteins are more sparsely distributed. Another approach has used matched-filter-based algorithms to circumvent the pitfalls of thresholding [8]. However, this method also relies on t-tubule regularity. Finally, although t-tubules and dyads are known to have complex configurations in three-dimensional space, available programs have until now been limited to two-dimensional analyses of these structures, and have not enabled examination of co-localization of dyadic proteins. Such analyses require not only careful thresholding of signals but also accurate methods for skeletonization.

Here, we developed an automated approach to analyse t-tubule and dyadic structures in images captured by confocal and Airyscan microscopy. The *Tubulator* software employs novel methods to circumvent the most common pitfalls occurring when analysing such data, while enabling quantification of the density, orientation and co-localization of t-tubule membranes and proteins. The current software is more versatile than previously published plugins, as it supports automated analysis of different cell types and multicellular preparations, and because it enables interpretation and reconstruction of the three-dimensional nature of dyadic structure.

## 2. Methods

### (a) Cardiomyocyte and cardiac tissue preparation

Animal experiments were approved by the ethics committee at the University of Oslo and performed in accordance with the Norwegian Animal Welfare Act and NIH Guidelines ((NIH publication no. 85-23, revised 2011). Wistar rat cardiomyocytes were isolated by retrograde Langendorff perfusion, and either imaged directly or fixated in 4% paraformaldehyde as previously described [9].

Methods for collection of human tissue were approved by the Regional Ethics Committee (project S-05172) in agreement with The Declaration of Helsinki and the Council of Europe Convention on Human Rights and Biomedicine. Left ventricular tissue was obtained from non-diseased donor hearts deemed unsuitable for transplant owing to surgical concerns. Tissue was snap-frozen in liquid nitrogen and, following cryosectioning (10–20 µm), fixed in 4% paraformaldehyde for 30 min (see [10] for details).

### (b) Labelling of t-tubules and dyads

Experimental imaging of t-tubules was performed by staining live cells with di-4-ANEPPS (Sigma-Aldrich), di-8-ANEPPS (Sigma-Aldrich), RH237 (ThermoFisher), CellMask (ThermoFisher) or FM1–43FX (ThermoFisher). In fixed cells and tissue sections, t-tubules were stained with wheat germ agglutinin conjugated to Alexa Fluor (488, 546 or 633). Dyadic proteins were labelled with primary antibodies against Caveolin-3 (Cav-3; Abcam, ab2912) and bridging integrator 1 (Bin-1; Santa Cruz, Sc-23918), and secondary antibodies coupled to Alexa Fluor 488 or 546 (ThermoFisher). Presented images of dyadic proteins and t-tubules were obtained using an LSM800 Airyscan confocal microscope (Zeiss, Jena, Germany) using a 63× magnification oil immersion objective. To test the versatility of the plugin, analysis of images captured with other confocal microscopes (Zeiss LSM 510 and 710) was also performed.

### (c) Set-up and usage of *Tubulator*

*Tubulator* was written in Matlab and compiled to a stand-alone application (https://gitlab.com/louch-group/tubulator-installer). The software requires installation of Matlab compiler runtime. The *Tubulator* executable file can then be copied to anywhere on a Windows PC. Technical details and limitations of *Tubulator* can be found in the electronic supplementary material.

### (d) Workflow of the software

**Figure 1.**
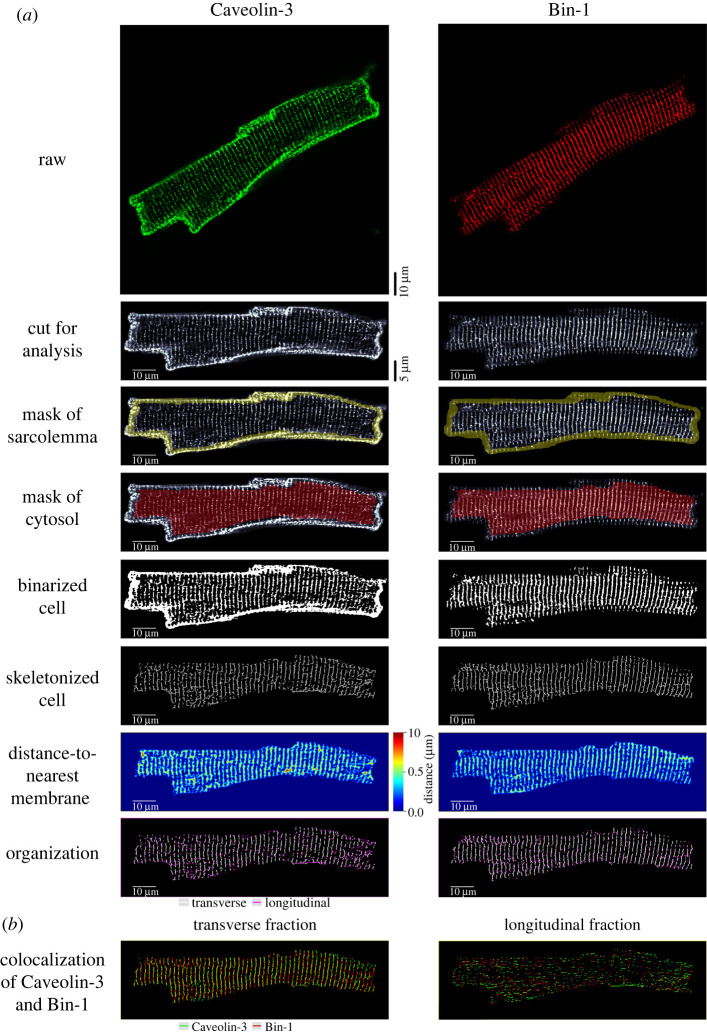
Overview of *Tubulator*'s workflow. (*a*) The individual stages of image processing are illustrated for a rat ventricular cardiomyocyte stained with antibodies against Caveolin-3 (green) and Bin-1 (red). First, the user is prompted to manually indicate the long axis of the cardiomyocyte, and roughly trace around the cell. *Tubulator* then automatically rotates the cell to align it within the analysis window. The sarcolemma and cytosol are then identified, and the image is binarized to enable quantification of the signal for the t-tubules or dyadic proteins of interest. These signals are then skeletonized to facilitate the creation of distance maps (the distance-to-nearest t-tubule/dyad or surface membrane) and signal separation into transverse and longitudinal components. (*b*) Images processed and printed by *Tubulator* provide an excellent basis for subsequent co-localization analysis of transverse and longitudinally oriented features.

A step-by-step user guide for *Tubulator* is provided in the electronic supplementary material. The workflow of the program is demonstrated in figure 1*a* for a representative, isolated rat ventricular cardiomyocyte immunolabelled for the dyadic proteins Caveolin-3 and Bin-1. Based on rough manual estimation of cell orientation and contours, *Tubulator* rotates and trims the raw image to fit and centre the cardiomyocyte in the analysis window. The ‘active contour' [11] function is then used to automatically define outer cellular borders and a sarcolemmal mask, to allow subsequent construction of a cytosolic mask. The bias that can occur when binarizing based on whole cell fluorescence values is circumvented by use of a custom-made adaptive threshold. This method of thresholding calculates local threshold levels across the cytosolic compartment, thereby reducing errors owing to uneven pixel intensity while ensuring that only structures of interest are highlighted. Based on the binarized image, *Tubulator* calculates the fraction of the cell covered by t-tubules or protein of interest.

Dyadic structures are examined in more detail, as the binarized image is skeletonized followed by morphological closing. Based on the resulting skeleton, distances to the skeleton are calculated for each pixel in the cytosol. These distances are then summarized in a ‘distance plot', illustrating the distance from all points in the cytosol to the nearest t-tubule or sarcolemmal membrane; a critical determinant of the synchrony of cellular Ca^2+^ release [12]. In addition, average, median and maximal intracellular distance and deviation are calculated and printed in a text file to provide more information about density and regularity of the examined structures. Lastly, *Tubulator* analyses the fractions of transverse and longitudinal elements by analysing the direction of neighbouring pixels in the skeleton.

**Figure 2.**
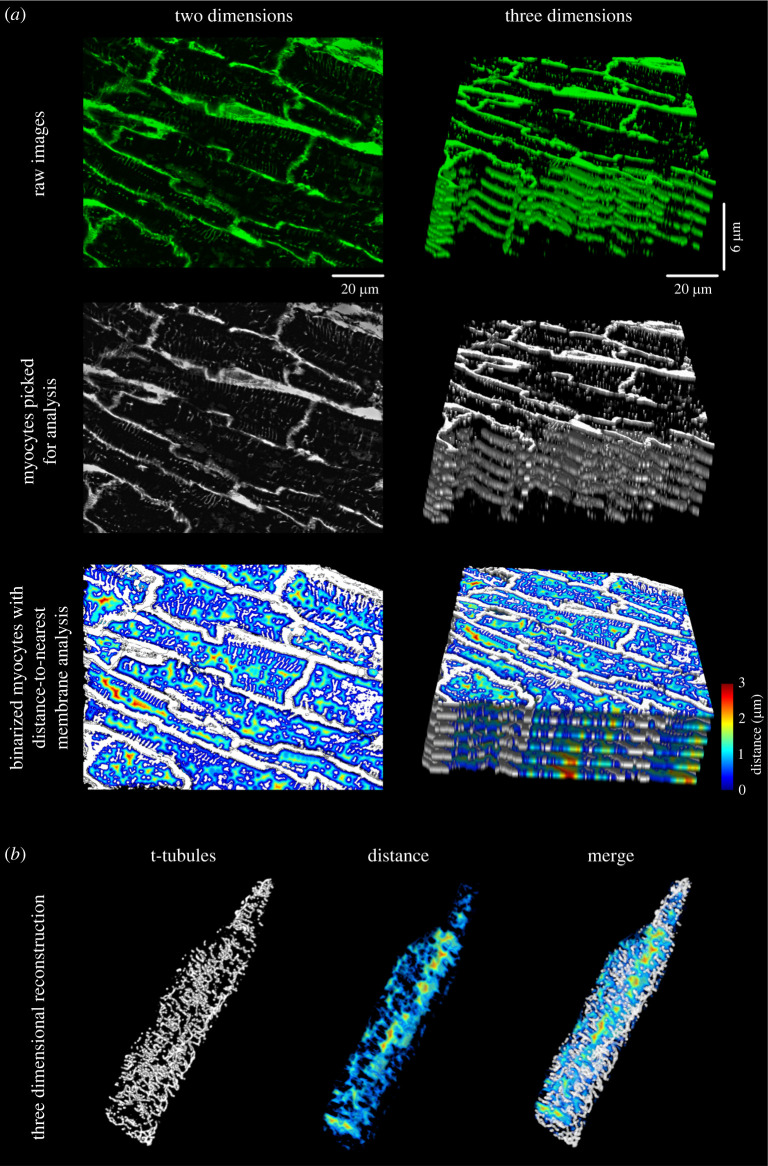
*Tubulator*'s versatility enables advanced analysis of tissue sections in three dimensions. (*a*) Human ventricular tissue section analysed in two dimensions (left) and three dimensions (right). *Tubulator* is able to perform detailed analysis on multiple cells from the same section in three dimensions. Each cell is analysed by the same methods as used for isolated cardiomyocytes (figure 1), including three-dimensional capabilities for distance map creation and rendering (*b*).

An important feature of *Tubulator* is the ability to analyse cells in tissue sections directly (figure 2*a*, left). The software enables analysis of several individual cells in each tissue section and can, provided that the analysed image is captured as a *z*-stack, also calculate distances to the nearest t-tubule or membrane in three-dimensional space (figure 2*a*, right). Three-dimensional reconstructions of the calculated metrics can subsequently be assembled using other programs (such as ImageJ, National Institute of Health, USA) as shown in figure 2*b*, and explained in more detail in [9].

### (e) Benchmarking *Tubulator's* performance

To test the accuracy and analytic power of *Tubulator*, an array of synthetic cardiomyocyte phantoms (*n* = 37 cells) was created in Matlab (Mathworks, Natick, MA, USA), with known t-tubule density and quantities of transverse and longitudinal elements. In brief, these synthetics cells were created using an image matrix consisting of 1024 × 1024 pixels, at a resolution of 6.67 pixels μm^−1^. In each matrix, the cell's surface membrane was simulated with a 900 × 200 pixel rectangle 7 pixels in thickness. Within this rectangle, transverse and longitudinal lines representing t-tubules were drawn. Transverse lines were spaced at intervals of 1.8 ± 0.3 µm, while longitudinal lines had an average distance of 5.6 ± 1.2 between them. To simulate a variable t-tubule density in both orientations, a random number generator was used to determine whether a given line would be drawn. The chance of losing a given line was set by the operator, varying from 0% to 100%. Exact densities of transverse and longitudinal tubules were calculated by counting the number of pixels containing transverse lines inside the cell, divided by the area of the cell. To simulate noise acquired during experimental imaging, a noise map was superimposed on simulated t-tubule images using random Gaussian noise created and filtered with convolution of a circular averaging filter. After analysing the final simulated images with *Tubulator*, comparison was made with the performance of previously published software [6,7].

## 3. Results and discussion

*Tubulator* was developed to improve upon existing methods for structural analysis of t-tubules and dyadic proteins (see [13–15]). Unlike previous contributions, the created software does not rely on regular structures for morphological analysis, and can be employed for examining the density, orientation and co-localization of t-tubule membranes and proteins in a variety of tissue preparations. *Tubulator* can also process and display image stacks to reveal detailed information of the three-dimensional nature of cardiomyocyte substructure. Finally, the program provides functional insight into the consequences of dyadic organization, by computing ‘distance maps' known to correlate with Ca^2+^ release synchrony and kinetics across the cell [12].

*Tubulator*'s adaptive thresholding method ensures binarization unbiased by cell contents (e.g. low or high signal density), while at the same time eliminating bias owing to variations in pixel intensity (electronic supplementary material, figure S1). Importantly, estimations of density are made prior to the skeletonization step, meaning that variations in t-tubule diameter are accounted for. The subsequent skeletonization step in the analysis pipeline enables extraction of key features of t-tubule/dyadic organization, allowing delineation of transversely and longitudinally oriented structures (figure 1*b*; electronic supplementary material, figure S2). Unlike currently available software, the technique used for these calculations is not based on FFT analysis, which is dependent on structural regularity, but rather determines the directionality of each pixel's neighbours.

**Figure 3.**
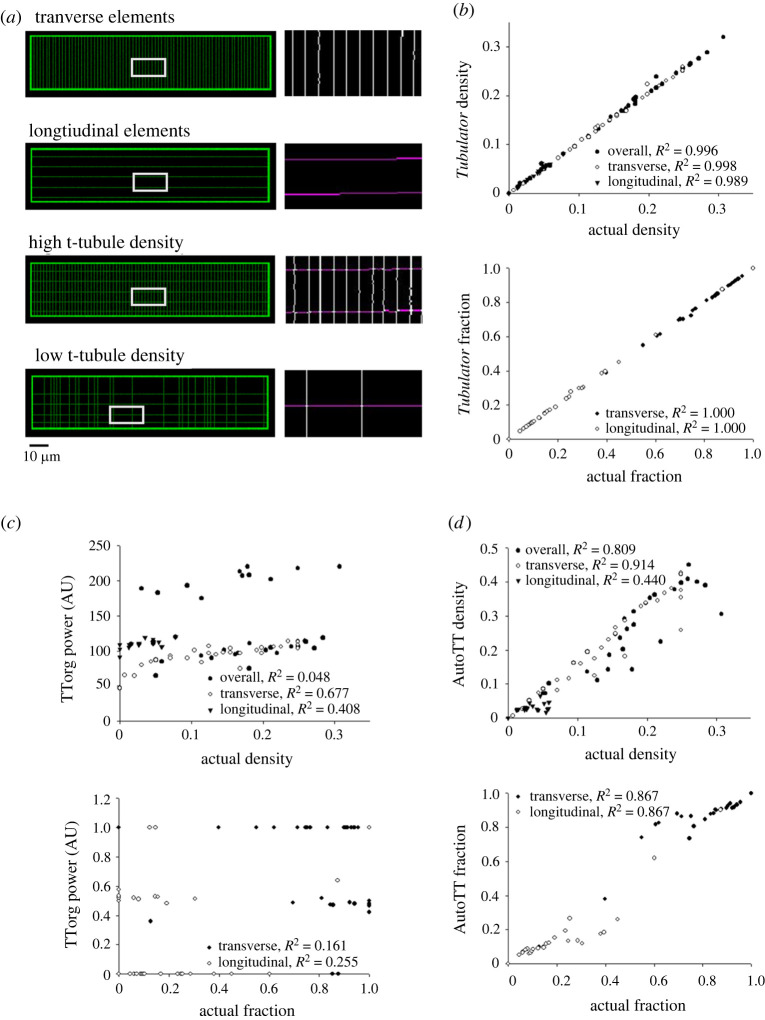
Benchmarking *Tubulator's* performance. Thirty-seven synthetic cardiomyocyte phantoms were created with known and variable densities of transverse and longitudinal tubules. (*a*) Representative synthetic cells with (from the top) only transverse elements, only longitudinal elements, a high density of both transverse and longitudinal elements, and low t-tubule density. Right panels display *Tubulator's* quantification of transverse (white) and longitudinal (purple) tubules in zoomed-in areas highlighted by the grey box. (*b*) We observed strong correlations between *Tubulator* outputs and actual t-tubule densities, including the proportion of transverse and longitudinal elements. (*c,d*) Correlations between actual t-tubule density and fractions measured with TTorg [6] and AutoTT [7], respectively. With these programs, outputs were not produced for six of the 31 simulated cells with the sparsest and least regular t-tubule arrangements.

To benchmark *Tubulator's* performance, synthetic cardiomyocyte phantoms were created, with t-tubule densities varying between 0% and 30% (figure 3*a*). Importantly, the images comprised differing compositions of transverse and longitudinal components. Example cells with high and low t-tubule density are shown in figure 3*a*. We observed that *Tubulator* accurately estimated t-tubule density, as outputs were strikingly similar to known values in the simulated images (figure 3*b*). Measurements of the densities and fractions of transverse and longitudinal elements were also found to be robust (figure 3*b*). To compare *Tubulator's* capabilities with previously published analysis tools (TTorg [6] and AutoTT [7]), the same analyses were performed using these programs (figure 3*c,d*, respectively). As expected, these macros were often unable to analyse cells with no transverse elements, or very low overall t-tubule density. Indeed, of the 37 cells analysed, TTorg and AutoTT failed to yield outputs for the six images with the sparsest and least regular t-tubule arrangements. This shortcoming highlights the pitfalls of traditional FFT-based analyses, and the advantage of not assuming the presence of regular structures. Notably, all programs estimated densities and fractions of transverse and longitudinal elements which were significantly correlated with actual values (figure 3*b–d*). However, *Tubulator* achieved the strongest associations, and the improved performance of *Tubulator* was not limited to cells with sparse t-tubules. Thus, the program is well suited for analysis of cardiomyocytes with a wide range of t-tubule or protein density.

As the arrangement of dyads in transverse and longitudinal orientations is frequently complex, greater insight is given by the produced distance maps. In addition to producing colour-coded images, average, median, and maximum distance and variability are conveyed to give a nuanced picture of both density and regularity of the analysed structures. For *z*-stacks, distances are additionally calculated in three-dimensional space to yield valuable information about the true three-dimensional nature of myocyte substructure.

*Tubulator* is also convenient to use for analysis of co-localization between proteins or structures of interest. The output image file contains both the binarized and skeletonized cell which can be readily employed for subsequent co-localization analyses. In addition, individual skeletons of transverse and longitudinal elements are produced, which provides a scaffold for assessing co-localization within these two orientations. As an example, a rat ventricular cardiomyocyte labelled with Bin-1 and Cav-3 is shown in figure 1*a*. Although previous reports have indicated that there is robust expression of these proteins along t-tubules [16], co-localization analysis enabled by *Tubulator* showed that Bin-1 and Cav-3 do not fully co-localize. Indeed, large fractions of transversely oriented Bin-1 were present in the absence of Cav-3 staining, and longitudinal co-localization was virtually non-existent (figure 1*b*).

In contrast to previously published software, *Tubulator* performs analyses in tissue sections directly (figure 2). The file structure outputted by *Tubulator* enables easy identification of individual cells' localization in a tissue section. This feature is especially useful when, for example, investigating regional differences in cardiac chambers [9,17]. Another obvious usage is whole heart *in situ* preparations, where individual cardiomyocytes can readily be selected for analysis [18,19].

## 4. Limitations

The main challenge for obtaining robust quantification of t-tubules and dyadic proteins is proper thresholding of staining signals. Here, a custom-made adaptive thresholding technique was developed to allow more accurate binarization. Although, this approach improves upon existing methods, it does not overcome all pitfalls of binarization entirely, and some risk of over- or under-estimating signal density remains. However, we have sought to minimize binarization bias by allowing the user to adjust the adaptive thresholding in the program. Importantly, our method relies on good image quality for optimal outputs and on rare occasions a different thresholding method may be preferred. Thus, *Tubulator* provides the possibility to analyse pre-binarized images, or binarization based on mean fluorescence.

*Tubulator* does not directly assess t-tubule widths, although previous work has shown that t-tubule dimensions may be significantly altered during cardiac disease [20]. Nevertheless, relative changes in t-tubule width can be estimated with *Tubulator* outputs, by comparing t-tubule densities between two groups before and after skeletonization. For example, we previously have shown that higher t-tubule density observed during heart failure with preserved ejection fraction was attributed to t-tubule dilation, since skeletonization reduced t-tubule signals to control values [15]. Alternatively, t-tubule widths may be assessed by simultaneously staining t-tubule membrane and lumen [21].

## 5. Conclusion

In summary, we developed software suited for detailed analysis of the three-dimensional organization of t-tubules and dyadic proteins, with easy application to isolated cardiomyocytes or tissue sections. We anticipate that *Tubulator*'s versatility and novel features for data extraction will aid researchers in interpreting data and generate new hypotheses. The software is freely available and can be accessed by contacting the authors.

## Data Availability

Matlab files and codes are available here: https://gitlab.com/louch-group/tubulator. The data are provided in the electronic supplementary material [22].
